# Efficacy and safety of chemoimmunotherapy in advanced non-small cell lung cancer patients with antibiotics-induced dysbiosis: a propensity-matched real-world analysis

**DOI:** 10.1007/s00432-024-05649-x

**Published:** 2024-04-26

**Authors:** Kentaro Tamura, Yusuke Okuma, Shogo Nomura, Akito Fukuda, Ken Masuda, Yuji Matsumoto, Yuki Shinno, Tatsuya Yoshida, Yasushi Goto, Hidehito Horinouchi, Noboru Yamamoto, Yuichiro Ohe

**Affiliations:** 1 Department of Thoracic Oncology, National Cancer Center Hospital, 5-1-1, Tsukiji, Chuo, Tokyo 104-0045 Japan; 2 Division of Respiratory Diseases, Department of Internal Medicine, The Jikei University School of Medicine, 3-25-8, Nishi-Shimbashi, Minato, Tokyo 105-8461 Japan; 3 Department of Biostatics and Bioinformatics, Graduate School of Medicine, The University of Tokyo, 7-3-1, Hongo, Bunkyo, Tokyo 113-8655 Japan; 4 Department of Experimental Therapeutics, National Cancer Center Hospital, 5-1-1, Tsukiji, Chuo, Tokyo 104-0045 Japan

**Keywords:** Antibiotics, Chemotherapy, Immune checkpoint inhibitors, Immune-related adverse event, Non-small cell lung cancer

## Abstract

**Purpose:**

The gut microbiota is hypothesized as a prognostic biomarker for cancer immunotherapy. Antibiotic-induced dysbiosis negatively affects the clinical outcomes of immunotherapy. However, the effect of dysbiosis on the efficacy and safety of Chemoimmunotherapy (chemo-IOs), the frontline standard of care, in advanced non-small cell lung cancer (NSCLC) remains unknown. We aimed to compare the efficacy and safety of chemo-IOs in patients exposed to antibiotics before treatment with those of patients who were not exposed.

**Methods:**

We retrospectively reviewed patients with advanced NSCLC treated with first-line chemo-IOs between 2018 and 2020 at the National Cancer Center Hospital. The patients were divided into two groups: those exposed to antibiotics within 30 days before induction therapy (ABx group) and those did not antibiotics (Non-ABx group). Propensity score matching was used to control for potential confounding factors. Clinical outcomes including progression-free survival (PFS), overall survival (OS), and immune-related adverse events (irAEs) were compared.

**Results:**

Of 201 eligible patients, 21 were in the ABx group, and 42 were in the non-ABx group after propensity score matching. No differences in PFS or OS emerged between the two groups (ABx group vs. Non-ABx group) (PFS:7.0 months vs. 6.4 months, hazard ratio [HR] 0.89; 95% confidence interval [CI], 0.49–1.63, OS:20.4 months vs. 20.1 months, HR 0.87; 95% CI 0.44–1.71). The frequency of irAEs before propensity score matching was similar across any-grade irAEs (39.4% vs. 42.9%) or grade 3 or higher irAEs (9.1% vs. 11.3%).

**Conclusion:**

Antibiotic-induced dysbiosis may not affect the efficacy of chemo-IOs in patients with advanced NSCLC.

**Supplementary Information:**

The online version contains supplementary material available at 10.1007/s00432-024-05649-x.

## Introduction

The standard of care for previously untreated advanced non-small cell lung cancer (NSCLC) is combined cancer immunotherapy (IO) and cytotoxic chemotherapy (chemo-IO) (Gandhi et al. 2018; Paz-Ares et al. 2018; Socinski et al. 2018; Mok et al. 2019; West et al. 2019; Nishio et al. 2021). Although programmed cell death ligand-1 (PD-L1) expression has been used as a biomarker to stratify prognosis in many trials, its usefulness in predicting IO response is limited. The CheckMate 227 study found that nivolumab plus ipilimumab was effective, regardless of PD-L1 expression levels (Hellmann et al. 2019). Thus, additional biomarkers are needed to predict the efficacy of IO and immune-related adverse events (irAEs) when selecting the most appropriate patients.

Gut microbiota plays a crucial role in maintaining host immune and metabolic homeostasis, and dysbiosis has been linked to various disorders (Nicholson et al. 2012). Studies have shown that specific gut microbiota species and diversity can affect IO efficacy and irAEs (Vétizou et al. 2015; Becattini et al. 2016; Korpela et al. 2016; Routy et al. 2018a; Soularue et al. 2018; Hakozaki et al. 2020). Therefore, the gut microbiota is a potential biomarker for predicting IO efficacy and safety. Antibiotics-induced dysbiosis has consistently been shown to have negative therapeutic effects on IO monotherapy in various types of cancer, including NSCLC (Wilson et al. 2020; Chalabi et al. 2020; Lurienne et al. 2020; Tsikala-Vafea et al. 2021; Cortellini et al. 2021a; Derosa et al. 2021). However, several retrospective studies mainly in Europe and North America have shown that antibiotic-induced dysbiosis does not negatively affect the therapeutic efficacy of chemo-IOs (Cortellini et al. 2021b; Hopkins et al. 2022). As the composition of the gut microbiota depends on geographic sites and dietary habits, it remains unclear whether antibiotic-induced dysbiosis influences the effectiveness and safety of chemo-IOs, particularly in Japan or other Asian countries (Derosa et al. 2021).

The purpose of this study was to investigate whether the use of antibiotics affects the efficacy and safety of chemo-IOs in the Japanese population and to determine whether dysbiosis prior to chemo-IOs can be used as a biomarker for predicting the efficacy and safety of Chemoimmunotherapy.

## Patients and methods

### Study design and patients

This retrospective cohort study enrolled consecutive patients with advanced NSCLC who underwent pembrolizumab or atezolizumab plus platinum-based chemotherapy between December 2018 and December 2020 at the National Cancer Center Hospital, Tokyo, Japan. Patients with oncogenic driver gene mutations after the failure of tyrosine kinase inhibitors were also included. Patients who had received cytotoxic chemotherapy previously or had participated in clinical trials were excluded. As antibiotics-induced dysbiosis within approximately 1 month of IOs administration was shown to impact treatment efficacy, we divided the patients into two groups: those treated with antibiotics within 30 days prior to induction therapy (ABx group) and those who did not undergo antibiotic treatment (Non-ABx group) (Wilson et al. 2020; Derosa et al. 2021).

### Data collection

Baseline patient characteristics prior to induction therapy, including PD-L1 status, and clinical outcomes, were collected from the electronic medical records (EMRs). The following information was also extracted: antibiotic type, route of administration, duration, and purpose of antibiotic treatment; concomitant medications such as proton pump inhibitors (PPIs), antihistamine blockers (H2B), and probiotics within 30 days of starting induction therapy; and factors related to antibiotic usages such as C-reactive protein (CRP) levels. Baseline maximum tumor size of the lung at the initiation of chemo-IOs was also investigated. PD-L1 expression levels were determined using a PD-L1 IHC 22C3 pharmDx (Agilent Technologies, Santa Clara, CA, USA). Tumor responses were evaluated according to the Response Evaluation Criteria in Solid Tumors (RECIST) criteria version 1.1 (Eisenhauer et al. 2009).

The attending physicians collected data on irAEs which were graded according to the National Cancer Institute Common Terminology Criteria for Adverse Events (CTCAE), version 5.0 (National Cancer Institute Cancer Therapy Evaluation Program (2017)). All irAEs observed from the date of starting Chemo-IOs to the cutoff date were collected from the EMRs. Data collection was conducted until the cutoff date of October 31, 2022. Structured data (predefined, formatted, and coded data) were extracted directly from the EMRs. Unstructured data (from written case notes) or data not directly extractable were collected by KT. All extracted data were downloaded for this study. Data collection depended on data availability, quality, and validity, assessed through data due diligence to ensure data conformity, completeness, and plausibility.

### Evaluation

The overall response rate (ORR) was defined as the percentage of patients with the best overall response either as a complete or partial response. Survival curves of progression-free survival (PFS) and overall survival (OS) were estimated using the Kaplan–Meier method. PFS was defined as the time from the start of induction therapy to disease progression, death due to any cause. Patients without progression were censored at the last follow-up. OS was defined as the time from the start of induction therapy to death due to any cause. Patients without death were censored at the last follow-up.

### Statistical analysis

Continuous variables such as age, maximum tumor size, and CRP level were dichotomized according to their median values.

The primary interest of this study is to examine whether an antibiotic-induced dysbiosis may be associated with patient outcomes. Considering potential confounding variables regarding ABx and non-ABx groups, a propensity score was estimated using a multivariable logistic regression model including histology, stage, smoking status, maximum tumor size, use of PPIs/H2B, and elevated CRP levels as covariates. Continuous variables including the maximum tumor size and CRP levels were dichotomized, and categorized by the median. The nearest neighbor 1:2 matching was employed with a caliper width of 0.2 standard deviations of the logit of the estimated propensity score.

For propensity score matched cohort, we performed univariable logistic regression for ORR and univariable Cox regression analysis for PFS and OS to estimate odds ratio and hazard ratios (HRs) with their 95% confidence intervals (CIs). As a benchmark, using propensity score unmatched cohort, their corresponding multivariable analyses were performed including the same covariates as used for estimating the propensity score.

All statistical analyses were conducted using EZR (Easy R) statistical software version 1.54 (Kanda 2013). All reported p-values were two-sided. This study was approved by the Committee of the National Cancer Center Hospital (2015–355). The opportunity to refuse this study by subjects was guaranteed with details of this study available to the public.

## Results

### Patient characteristics

**Fig. 1:**
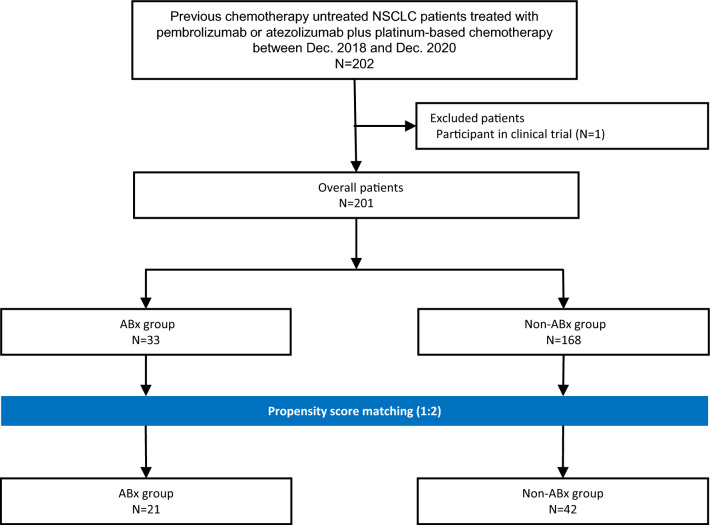
Study flow diagram. ABx group, patients treated with antibiotics 30 days before induction therapy; chemo-IOs, combined cytotoxic chemotherapy and cancer immunotherapy; Non-ABx group, patients treated without antibiotics 30 days before induction therapy; PSM, propensity score matching

Of the 202 patients who underwent first-line chemo-IO for advanced NSCLC, 201 were included in this study (Fig. 1). Prior to propensity score matching, 33 patients (15.8%) were in the ABx group and 168 (80.8%) were in the non-ABx group. After propensity score matching (PSM), 21 and 42 patients were included in the ABx and non-ABx groups, respectively.

Baseline patient characteristics are summarized in Table 1. In the overall cohort prior to PSM, the median age was 66.0 years (interquartile range [IQR] 58.0–71.0), 67.7% of patients were male, 93.0% had PS 0–1, and 78.1% were smokers. Eighty (39.8%) patients used PPIs/H2B and 5 (2.5%) patients used probiotics. Compared to the non-ABx group, the ABx group had more patients with squamous cell carcinoma ([ABx group vs. Non-ABx group] 42.4% vs. 13.7%), tumor size of 40 mm or larger (72.7% vs. 38.1%), and elevated CRP (≥ 1.04 mg/dl) (75.8% vs. 45.2%). Baseline variables used for estimating propensity score were tended to be well balanced between the two groups.

**Table 1:** Baseline patient characteristics

n (%)	Before propensity score matching				After propensity score matching		
	Total (n = 201)	ABx (n = 33)	Non-ABx (n = 168)	Std diff	ABx (n = 21)	Non-ABx (n = 42)	Std diff
Age, median (IQR)	66.00 (58.00, 71.00)	66.00 (62.00, 72.00)	65.50 (56.75, 71.00)	0.295	65.00 (62.00, 71.00)	67.00 (60.25, 69.75)	0.393
≥ 70, n (%)	66 (32.8)	13 (39.4)	53 (31.5)	0.165	6 (28.6)	11 (26.2)	0.053
Sex							
Male/female	136 (67.7)/65 (32.3)	26 (78.8)/7 (21.2)	110 (65.5)/58 (34.5)	0.300	17 (81.0)/4 (19.0)	31 (73.8)/11 (26.2)	0.171
ECOG-PS							
0–1/2	187 (93.0)/14 (7.0)	32 (97.0)/1 (3.0)	155 (92.3)/13 (7.7)	0.210	20 (95.2)/1 (4.8)	40 (95.2)/2 (4.8)	< 0.001
Smoking status							
Never/Ex or Current	44 (21.9)/157 (78.1)	3 (9.1)/30 (90.9)	41 (24.4)/127 (75.6)	0.419	2 (9.5)/19 (90.5)	1 (2.4)/41 (97.6)	< 0.001
Histological subtype							
Non-SQC/SQC	164 (81.6)/37 (18.4)	19 (57.6)/14 (42.4)	145 (86.3)/23 (13.7)	0.675	16 (76.2)/5 (23.8)	34 (81.0)/8 (19.0)	0.116
Stage							
III/IV or Recurrence	14 (7.0)/187 (93.0)	5 (15.2)/28 (84.8)	9 (5.4)/159 (94.6)	0.327	1 (4.8)/20 (95.2)	2 (4.8)/40 (95.2)	< 0.001
Maximum tumor size at the start of Chemoimmunotherapy							
Median (IQR) (mm)	42.0 (26.0, 60.0)	40.0 (24.0, 57.5)	58.0 (44.0, 72.3)	0.714	55.0 (40.5, 66.0)	58.0 (44.0, 64.0)	0.173
< 40 mm/ ≥ 40 mm/NE	65 (32.3)/88 (43.8)/48 (23.9)	6 (18.2)/24 (72.7)/3 (9.1)	59 (35.1)/64 (38.1)/45 (26.8)	0.618	4 (19.0)/17 (81.0)/0 (0.0)	8 (19.0)/34 (81.0)/0 (0.0)	< 0.001
Oncogenic driver gene mutation^a^	24 (11.9)	1 (3.0)	23 (13.7)	0.392	1 (4.8)	5 (11.9)	0.186
PD-L1 TPS							
< 1/1–49/50– (%)	77 (38.3)/61 (30.3)/55 (27.4)	16 (48.5)/8 (24.2)/9 (27.3)	61 (36.3)/53 (31.5)/46 (27.4)	0.394	10 (47.6)/4 (19.0)/7 (33.3)	14 (33.3)/14 (33.3)/11 (26.2)	0.402
NE	8 (4.0)	0 (0.0)	8 (4.8)		0 (0.0)	3 (7.1)	
Liver metastases	21 (10.4)	2 (6.1)	19 (11.3)	0.187	1 (4.8)	7 (16.7)	0.261
CRP, median (IQR) (mg/dl)	1.04 (0.15, 3.79)	0.80 (0.13, 3.03)	4.57 (1.04, 9.14)	0.745	2.60 (1.11, 4.55)	3.77 (1.02, 8.66)	0.418
CRP ≥ 1.04 (mg/dl)	101 (50.2)	25 (75.8)	76 (45.2)	0.657	15 (71.4)	32 (76.2)	0.108
Regimen				0.632			0.161
CBDCA or CDDP + PEM + Pembro or Atezo	138 (68.6)	17 (51.5)	121 (72.0)		15 (71.4)	26 (61.9)	
CBDCA + PTX/nab-PTX + Pembro or Atezo	46 (22.9)	15 (45.5)	31 (18.5)		5 (23.8)	12 (28.6)	
CBDCA + PTX + Beva + Atezo	17 (8.5)	1 (3.0)	16 (9.5)		1 (4.8)	4 (9.5)	
Treatment line							
1/ ≥ 2	186 (92.5)/15 (7.5)	33 (100.0)/0 (0.0)	153 (91.1)/15 (8.9)	0.443	21 (100.0)/0 (0.0)	39 (92.9)/3 (7.1)	0.545
Use of PPIs/H_2_B	80 (39.8)	13 (39.4)	67 (39.9)	0.010	8 (38.1)	17 (40.5)	0.100
Use of probiotics	5 (2.5)	1 (3.0)	4 (2.4)	0.040	1 (4.8)	1 (2.4)	0.129

ABx group, patients treated with antibiotics 30 days prior to induction therapy; Atezo, atezolizumab; Beva, bevacizumab; CBDCA, carboplatin; CDDP, cisplatin; Chemo-IO, combined cytotoxic chemotherapy and cancer immunotherapy; CRP, C-reactive protein; ECOG-PS, Eastern Cooperative Oncology Group-performance status; IQR, interquartile range; nab-PTX, nab-paclitaxel; NE, not evaluated; Non-ABx group, patients treated without antibiotics 30 days prior to induction therapy; Non-SQC, non-squamous cell carcinoma; PD-L1, programmed cell death-ligand 1; PEM, pemetrexed; Pembro, pembrolizumab; PTX, paclitaxel; PPIs/H2B, proton pump inhibitors/antihistamine blockers; Std diff, standardized difference; SQC, squamous cell carcinoma; TPS, tumor proportion score

^a^Oncogenic driver mutations include *EGFR*, *ALK*, *ROS-1*, *BRAF*, *MET* exon 14 skipping, *RET*, and *NTRK*

In the ABx group, prophylaxis was the most common reason for using antibiotics ([before PSM] 54.5%, [after PSM] 52.6%), particularly for pneumonia during bronchoscopy ([before PSM] 42.4%, [after PSM] 42.9%) (Table 2). Pneumonia was the most common cause for curative antibiotic therapy ([before PSM] 33.3%, [after PSM] 42.9%). Most patients were prescribed anaerobic-spectrum agents ([before PSM] 78.8%, [after PSM] 85.7%), including penicillin and beta-lactamase inhibitor combinations ([before PSM] 75.8%, [after PSM] 84.2%).

**Table 2:** Details of antibiotic usage

n (%)	Before PSM (n = 33)	After PSM (n = 21)
Purposes for using antibiotics^a^		
Prophylaxis for pneumonia at BF	14 (42.4)	9 (42.9)
Pneumonia	11 (33.3)	9 (42.9)
Prophylaxis for sepsis at extracting teeth	2 (6.1)	1 (4.8)
Chronic bronchitis	2 (6.1)	0 (0.0)
Prophylaxis for PCP at the use of corticosteroids	2 (6.1)	1 (4.8)
Urinary tract infection	1 (3.0)	1 (4.8)
Fever	1 (3.0)	1 (4.8)
Cellulitis	1 (3.0)	0 (0.0)
Type of antibiotics^a^		
AMPC/CVA	21 (63.6)	14 (66.7)
PIPC/TAZ	5 (15.2)	3 (14.3)
LVFX	4 (12.1)	3 (14.3)
ABPC/SBT	3 (9.1)	3 (14.3)
ST	2 (6.1)	1 (4.8)
CAM	2 (6.1)	0 (0.0)
AMPC	1 (3.0)	0 (0.0)
CEZ	1 (3.0)	0 (0.0)
MFLX	1 (3.0)	1 (4.8)
VCM	1 (3.0)	0 (0.0)
Anaerobic-spectrum agents	26 (78.8)	18 (85.7)
Duration of antibiotics use ≥ 1 week	20 (60.6)	12 (57.1)
Route of antibiotics^a^		
Intravenous/oral	9 (27.3)/28 (84.8)	6 (28.6)/18 (85.7)

AMPC, amoxicillin; AMPC/CVA, amoxicillin/clavulanate; BF, bronchofiberscopy; CAM, clarithromycin; CEZ, cefazolin; MFLX, moxifloxacin; PCP, Pneumocystis pneumonia; PIPC/TAZ, piperacillin/tazobactam; PSM, propensity score matching; ST, sulfamethoxazole-trimethoprim; VCM, vancomycin

^a^Patients were counted in more than one row

### Efficacy following propensity score matching

**Fig. 2:**
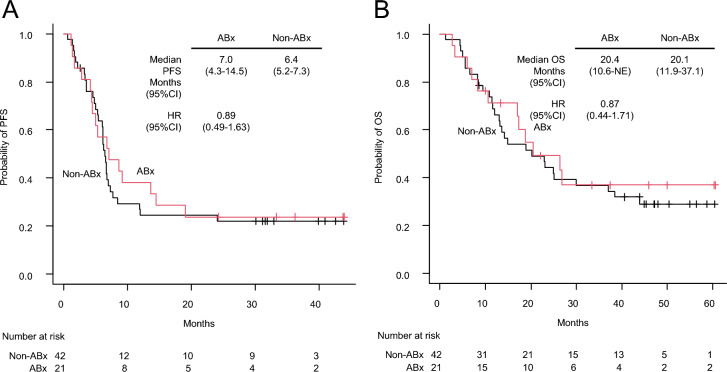
Survival efficacy following propensity score matching. Kaplan–Meier estimates of progression-free survival (PFS) (**A**) and overall survival (OS) (**B**) between patients treated with antibiotics 30 days prior to induction therapy (ABx group) and those who did not receive antibiotics (Non-ABx group) after propensity score matching. CI, confidence interval; HR, hazard ratio

**Fig. 3:**
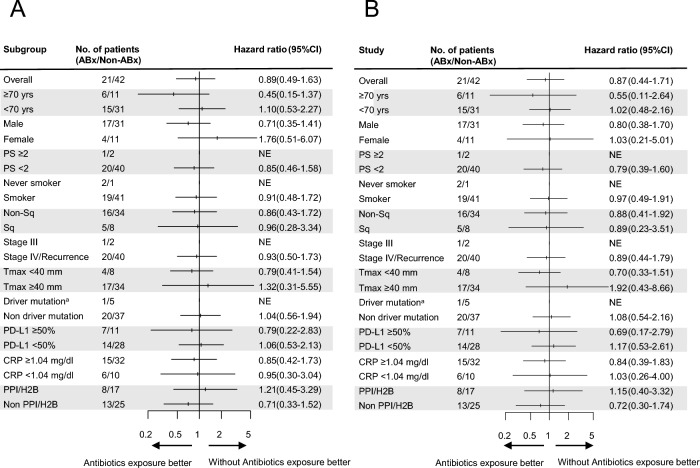
Subgroup analysis for survival efficacy following propensity score matching. Forest plots showing PFS (**A**) and OS (**B**) between patients treated with antibiotics 30 days before induction therapy (ABx group) and those who did not receive antibiotics (Non-ABx group) according to subgroup. ^a^Oncogenic driver mutations include *EGFR*, *ALK*, *ROS-1*, *BRAF*, *MET* exon 14 skipping, *RET*, and *NTRK*. CI, confidence interval; CRP, C-reactive protein; NE, not evaluated; Non-SQC, non-squamous cell carcinoma; PD-L1, programmed cell death-ligand 1; PPI/H2B, proton pump inhibitors/antihistamine blockers; PS, Eastern Cooperative Oncology Group performance status; SQC, squamous cell carcinoma; Tmax, maximum tumor size at the start of induction therapy

The ABx group had a median follow-up period of 45.9 months, while the non-ABx group had a median follow-up period of 47.2 months. The ORR showed no difference between the two groups (52.1% [95% CI 29.8–74.3] vs. 45.2% [95% CI 29.8–61.3%]) (Supplementary Table S1). The median PFS and OS were also not different between the two groups (PFS:7.0 months vs. 6.4 months, HR 0.89 [95% CI:0.49–1.63]; OS:20.4 months vs. 20.1 months, HR 0.87 [95% CI:0.44–1.71]) (Fig. 2a, b). Subgroup analyses for PFS and OS showed no differences among the baseline patient characteristics (Fig. 3a, b). Subgroup analysis of the ABx group based on the administration route (intravenous vs. oral), duration (≥ 1 week vs. < 1 week), spectrum (anaerobic vs. non-anaerobic), and purpose (curative vs. prophylaxis) of antibiotics did not reveal any differences in PFS or OS between the two groups (Supplementary Fig. S1).

### Multivariable analysis of efficacy prior to propensity score matching

We conducted a multivariable analysis of the cohort before PSM for ORR, PFS, and OS (Supplementary Table S2). There was no difference between the ABx group and the non-ABx group (ORR: Odds ratio 0.69 [95% CI 0.28–1.71], PFS: HR 0.91 [95% CI 0.56–1.49], OS: HR 0.99 [95% CI 0.57–1.73]). Elevated CRP (≥ 1.04 mg/dl) was a factor for worse PFS and OS (PFS: HR 2.30 [95% CI 1.49–3.55], OS: HR 3.00 [95% CI 1.80–5.01]).

### Immune-related adverse events before propensity score matching

There was no difference in the frequency of irAEs between the ABx and non-ABx groups (39.4% vs. 42.9%) (Table 3). There was also no difference in the frequency of grade 3 or higher irAEs between the two groups (9.1% vs. 11.3%). Although the frequencies of interstitial lung disease, colitis, and skin reactions were higher in the non-ABx group compared to the ABx group, the frequency of grade 3 or higher serious adverse events did not differ between the two groups.

**Table 3:** Immune-related adverse events before propensity score matching

	ABx (n = 33)	Non-ABx (n = 168)
Any grade	13 (39.4)	72 (42.9)
Grade 3–5	3 (9.1)	19 (11.3)

	ABx (n = 33)		Non-ABx (n = 168)	
	Any grade	Grade 3–5	Any grade	Grade 3–5
Interstitial lung disease	6 (18.2)	1 (3.0)	13 (7.7)	8 (4.8)
Colitis	5 (15.2)	1 (3.0)	16 (9.5)	2 (1.2)
Skin reaction	3 (9.1)	0 (0.0)	12 (7.1)	3 (1.8)
Cholangitis	1 (3.0)	1 (3.0)	3 (1.8)	2 (1.2)
AST elevated	1 (3.0)	0 (0.0)	0 (0.0)	0 (0.0)
Encephalitis	1 (3.0)	0 (0.0)	0 (0.0)	0 (0.0)
Hypothyroidism	0 (0.0)	0 (0.0)	14 (8.3)	0 (0.0)
Hypoadrenalism	0 (0.0)	0 (0.0)	10 (6.0)	0 (0.0)
Renal insufficiency	0 (0.0)	0 (0.0)	4 (2.4)	1 (0.6)
Stomatitis	0 (0.0)	0 (0.0)	3 (1.8)	0 (0.0)
Fever	0 (0.0)	0 (0.0)	2 (1.2)	0 (0.0)
Cellulitis	0 (0.0)	0 (0.0)	1 (0.6)	0 (0.0)
Infusion related reaction	0 (0.0)	0 (0.0)	1 (0.6)	0 (0.0)
Pericarditis	0 (0.0)	0 (0.0)	1 (0.6)	1 (0.6)

ABx group, patients treated with antibiotics 30 days before induction therapy; AST, aspartate aminotransferase; Non-ABx group, patients treated without antibiotics 30 days before induction therapy

## Discussion

Exposure to antibiotics within 30 days prior to the administration of chemo-IOs did not affect efficacy or safety. Although the group exposed to antibiotics may represent a population with a poor prognosis, similar results were obtained after adjusting for background factors. The reproducibility of the results was confirmed by the duration and route of administration of antibiotics, as well as anaerobic-spectrum antibiotics.

Antibiotics not only kill pathogens but also disrupt the diversity and composition of the gut microbiota, thereby altering the immune environment (Morgun et al. 2015). To succeed in cancer IO, it is important to accelerate the cancer immune cycle (Chen and Mellman 2013). Although the mechanism by which the gut microbiota affects the tumor immune environment is complex and not yet fully understood, tumor antigenicity and adjuvanticity are associated with antitumor immune responses. Antigenicity allows antigen mimicry between bacterial and tumor antigens to prime antitumor T cells (Zitvogel et al. 2016; Derosa et al. 2021). Adjuvanticity shows that pathogen recognition receptors, also called pattern recognition receptors, are activated by the microbiome and stimulate cytokines and interferons, resulting in the modulation of the immune tonus (Zitvogel et al. 2016; Derosa et al. 2021). These mechanisms facilitate the cancer immunity cycle. However, not all bacteria in the gut microbiome are associated with antitumor immune responses. Specific bacterial species, including *Akkermansia muciniphila*, *Bacteroides fragilis*, *Bifidobacterium* spp., *Collinsella* spp., and *Ruminococcas* spp., and their diversity and differential abundance are beneficial to the tumor immune environment (Vétizou et al. 2015; Matson et al. 2018; Routy et al. 2018b; Hakozaki et al. 2020). Antibiotics eliminate these beneficial bacteria and reduce their diversity, which has negative clinical effects on IOs. Indeed, the use of antibiotics before anti-PD-1/PD-L1 or anti-CTLA4 antibody monotherapy was associated with negative clinical outcomes in several meta-analyses and prospective studies, regardless of cancer type and geographic region (Lurienne et al. 2020; Wilson et al. 2020; Derosa et al. 2021; Matson et al. 2018).

Cytotoxic chemotherapy induces immunogenic cell death via neoantigens released from direct damage to cancer cells, resulting in enhanced efficacy when combined with IO successfully (Galluzzi et al. 2015). The gut microbiota stimulates immune cells from the bone marrow to produce reactive oxygen species (Iida et al. 2013). As a result, oxidative stress in tumors enhances DNA damage by platinum-based agents (Iida et al. 2013). Mice treated with antibiotics show reduced antitumor responses (Iida et al. 2013). Although these results suggest that antibiotic exposure before chemo-IOs may negatively impact clinical outcomes, our results are contradictory. Cytotoxic chemotherapy disrupts the gut mucosal barrier and alters the gut microbiota (Viaud et al. 2013). Although the underlying mechanism remains unclear, chemotherapy may overcome its negative effects. The addition of chemotherapy to IO may counteract the negative effects of antibiotic exposure. In the clinical setting, a European multicenter retrospective study supported our results that antibiotic exposure before chemo-IOs had no effect on efficacy (Cortellini et al. 2021b). Our results are the first to suggest that the efficacy of chemo-IOs does not depend on the gut microbiota in the Japanese population.

The gut microbiota is responsible for the occurrence of irAEs, which are characterized as IO toxicities. Although the mechanism remains unknown, activated T cells react mutually with tumors and healthy organ antigens and may be associated with increased levels of pre-existing autoantibodies, increased levels of inflammatory cytokines, and enhanced complement-mediated inflammation due to the direct connection of cytotoxic antibodies (Postow et al. 2018). The impact of gut microbiota on tumor immunity is similar to that of irAEs in terms of antigenicity. It is noteworthy that the administration of antibiotics before IOs has been shown to disrupt the gut microbiota and subsequently reduce the incidence of irAEs (Kostine et al. 2021; Neo et al. 2022). Certain bacterial species, such as *Bacteroides spp.*, have been found to promote anti-tumor immune responses and confer resistance to immune-related colitis (Dubin et al. 2016). Although the use of cytotoxic chemotherapy enhances the effectiveness of IOs, the administration of antibiotics before chemotherapy may increase the risk of irAEs. Although the underlying mechanism is unclear, genetic factors such as HLA-A have been linked to irAEs, and various other factors may also contribute to their occurrence (Dubin et al. 2016). However, our results do not allow us to draw any conclusions regarding the safety impact of antibiotic exposure prior to chemo-IOs administration.

This study has several limitations. First, the retrospective, single-center design of this study restricts the generalizability of the results to other populations. Although the intestinal environment is significantly influenced by environmental and dietary habits, these factors cannot be determined from medical records. Additionally, patients who grew up abroad were underrepresented in our Japanese cancer center; thus, these findings should be generalized to other populations with caution. Second, the sample size of patients treated with probiotics was too small to draw conclusions regarding their efficacy in restoring antitumor responses (Tomita et al. 2020). Third, although propensity score matching and multivariable analysis were used to adjust for confounding factors, the use of antibiotics may have been associated with a poorer prognosis in certain populations. However, due to the small sample size, these strategies cannot adjust for all potential confounding factors in order to obtain reliable estimates of the hazard ratios for the PFS and OS. Fourth, the fecal microbiota was not identified using metagenomic analysis, which could have further clarified the role of the gut microbiota in this context. Further studies using metagenomic analyses are currently underway in Japan (Shiraishi et al. 2022). Finally, the CheckMate 9LA regimen, a combination of immunotherapy and cytotoxic chemotherapy, was excluded from the analysis because of its unavailability at the time of the study (John et al. 2022).

## Conclusion

In conclusion, our study indicates that the administration of antibiotics within 30 days before chemo-IOs does not seem to significantly affect the effectiveness of treatment in the Japanese population.

## Supplementary Information

Below is the link to the electronic supplementary material.

Supplementary file 1 (DOCX 93 kb)

## Data Availability

The datasets analyzed during the current study are available from the corresponding author on reasonable request.

## Abbreviations

ABx: Antibiotics
chemo-IO: Combined cytotoxic chemotherapy and cancer immunotherapy
Cis: Confidence intervals
CRP: C-reactive protein
CTCAE: Common Terminology Criteria for Adverse Events
EMRs: Electronic medical records
HRs: Hazard ratios
H2B: Anti-histamine blockers
IO: Cancer immunotherapy
IQR: Interquartile range
irAEs: Immune-related adverse events
NSCLC: Non-small cell lung cancer
ORR: Overall response rate
OS: Overall survival
PD-L1: Programmed cell death ligand-1
PFS: Progression-free survival
PPI: Proton pump inhibitors
PSM: Propensity score matching
RECIST: Response Evaluation Criteria in Solid Tumors
