# Supplementary material for: Efficacy and safety of chemoimmunotherapy in advanced non-small cell lung cancer patients with antibiotics-induced dysbiosis: a propensity-matched real-world analysis
Source: J Cancer Res Clin Oncol. 2024 Apr 26;150(4):216. doi: 10.1007/s00432-024-05649-x (PMC11052849; doi:10.1007/s00432-024-05649-x)
Supplement: Supplementary file 1 — (DOCX 93 kb) [file 432_2024_5649_MOESM1_ESM.docx]

**Supplementary Figure**

**
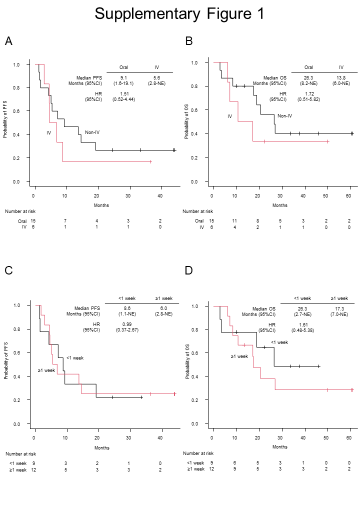
**

**
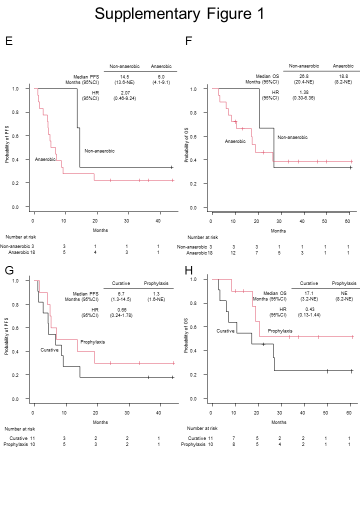
**

**Figure 1 Subgroup analysis of survival efficacy in patients with antibiotics exposure 30 days prior to induction therapy**

Kaplan-Meier estimates of progression-free survival (PFS) and overall survival (OS) in a patient with antibiotics exposure 30 days prior to induction therapy based on the route (intravenous vs. oral) (A: PFS, B: OS), duration (≥1 week vs. < 1 week) (C: PFS, D: OS), spectrum (anaerobic vs. non-anaerobic) (E: PFS, F: OS), and purpose (curative vs. prophylaxis) (G: PFS, H: OS).

CI, confidence interval; HR, hazard ratio; IV, intravenous; NE, not evaluated.

**Supplementary Table 1 Best responses following propensity score matching**

|  | Overall (n=63) | ABx (n=21) | Non-ABx (n=42) |
| --- | --- | --- | --- |
| Best overall responses, n (%) |  |  |  |
| Complete response | 0 (0.0) | 0 (0.0) | 0 (0.0) |
| Partial response | 30 (47.6) | 11 (52.4) | 19 (45.2) |
| Stable disease | 20 (31.7) | 6 (28.6) | 14 (33.3) |
| Progressive disease | 12 (19.0) | 3 (14.3) | 9 (21.4) |
| Not evaluated | 1 (1.6) | 1 (4.8) | 0 (0.0) |
| Overall response rate | 30 (47.6) | 11 (52.4) | 19 (45.2) |

ABx group, patients treated with antibiotics 30 days prior to induction therapy; non-ABx group, patients treated without antibiotics 30 days prior to induction therapy.

**Supplementary Table 2 Multivariable analysis of efficacy before propensity score matching**

|  | Overall response rate | Progression-free survival | Overall survival |
| --- | --- | --- | --- |
|  | Odds ratios (95%CI) | Hazard ratios (95%CI) | Hazard ratios (95%CI) |
| Antibiotics | | | |
| ABx vs. Non-ABx | 0.69 (0.28-1.71) | 0.91 (0.56-1.49) | 0.99 (0.57-1.73) |
| Smoking status | | | |
| Smoker vs. Never | 0.72 (0.30-1.72) | 0.62 (0.38-1.02) | 0.63 (0.35-1.16) |
| Histology | | | |
| SQC vs Non-SQC | 1.21 (0.48-3.07) | 1.27 (0.78-2.07) | 1.57 (0.92-2.67) |
| Stage | | | |
| IV/recurrence vs. III | 2.52 (0.74-8.62) | 1.23 (0.63-2.40) | 1.03 (0.49-2.18) |
| Maximum tumor size at the start of Chemo-IO | | | |
| ≥40 mm vs <40 mm | 1.00 (0.50-2.00) | 0.99 (0.67-1.46) | 1.09 (0.69-1.70) |
| CRP | | | |
| ≥1.04 mg/dl vs <1.04 mg/dl | 1.70 (0.82-3.53) | 2.30 (1.49-3.55) | 3.00 (1.80-5.01) |
| Use of PPIs/H2B | | | |
| Yes vs. No | 1.11 (0.57-2.19) | 1.05 (0.72-1.53) | 1.21 (0.80-1.84) |

ABx group, patients treated with antibiotics 30 days before induction therapy; Chemo-IO, combined cytotoxic chemotherapy and cancer immunotherapy; CI, confidence interval; CRP, C-reactive protein; Non-ABx group, patients treated without antibiotics 30 days before induction therapy; Non-SQC, non-squamous cell carcinoma; PPIs/H2B, proton pump inhibitors/antihistamine blockers; SQC, squamous cell carcinoma.
